# Novel transcriptional regulation of VEGF in inflammatory processes

**DOI:** 10.1111/jcmm.12020

**Published:** 2013-02-18

**Authors:** Xiaoren Tang, Yu Yang, Huaiping Yuan, Jian You, Marina Burkatovskaya, Salomon Amar

**Affiliations:** a Center for Anti-Inflammatory Therapeutics, Boston University Goldman School of Dental MedicineBoston, MA, USA; b Division of Oral and Maxillo-Facial Surgery, Tel Aviv Sourasky Medical CenterTel Aviv, Israel

**Keywords:** regulation, VEGF, LITAF, STAT6B, interaction, angiogenesis

## Abstract

Vascular endothelial growth factor (VEGF) is a critical angiogenic factor affecting endothelial cells, inflammatory cells and neuronal cells. In addition to its well-defined positive role in wound healing, pathological roles for VEGF have been described in cancer and inflammatory diseases (*i.e*. atherosclerosis, rheumatoid arthritis, inflammatory bowel disease and osteoarthritis). Recently, we showed that transcription factors LITAF and STAT6B affected the inflammatory response. This study builds upon our previous results in testing the role of mouse LITAF and STAT6B in the regulation of VEGF-mediated processes. Cells cotransfected with a series of VEGF promoter deletions along with truncated forms of mLITAF and/or mSTAT6B identified a DNA binding site (between −338 and −305 upstream of the transcription site) important in LITAF and/or STAT6B-mediated transcriptional regulation of VEGF. LITAF and STAT6B corresponding protein sites were identified. In addition, siRNA-mediated knockdown of mLITAF and/or mSTAT6B leads to significant reduction in VEGF mRNA levels and inhibits LPS-induced VEGF secretion in mouse RAW 264.7 cells. Furthermore, VEGF treatment of mouse macrophage or endothelial cells induces LITAF/STAT6B nuclear translocation and cell migration. To translate these observations *in vivo*, VEGF164-soaked matrigel were implanted in whole-body LITAF-deficient animals (TamLITAF^−/−^), wild-type mice silenced for STAT6B, and in respective control animals. Vessel formation was found significantly reduced in TamLITAF^−/−^ as well as in STAT6B-silenced wild-type animals compared with control animals. The present data demonstrate that VEGF regulation by LITAF and/or STAT6B is important in angiogenesis signalling pathways and may be a useful target in the treatment of VEGF diseases.

## Introduction

Inflammation is a protective response mediated by both innate and adaptive arms of the immune system following exposure to a range of harmful stimuli. Although inflammation is an essential mechanism in response to challenges including tissue injury and microbiological insult, inappropriate or excessive induction of the inflammatory response is itself a well-characterized cause of morbidity and mortality in adult populations contributing to pathologic conditions including autoimmune disorders [1–4], cancer [5, 6] and cardiovascular diseases [7–10]. There is currently a growing appreciation of the potential for inflammation to play an adverse role in tissue health. The expression of cytokines [notably interleukin 1β (IL-1β), IL-6, IL-8 and tumour necrosis factor alpha (TNF-α)] by tissue has been demonstrated to up-regulate the activity of a number of factors [*e.g*. prostaglandin hormones and their receptors, matrix metalloproteinases and vascular endothelial growth factor (VEGF)].

Vascular endothelial growth factor, a critically important mediator of vasculogenesis, is a homodimeric 34–42 kD heparin-binding glycoprotein. The VEGF family consists of five members, the most important of which is VEGF-A, previously referred as VEGF. The other members are placenta growth factor (PlGF), VEGF-B, VEGF-C and VEGF-D. These members are known to bind to VEGFR1/2 receptor that is implicated in all aspects of normal and pathological vascular endothelial cell biology [11]. VEGF is also known to play a central role in inflammation and wound healing by controlling both angiogenesis and vascular permeability. In addition, VEGF can affect other aspects related to inflammation such as induce osteoblast differentiation during bone repair processes [12]. However, much of the impetus to characterize the actions and regulation of VEGF stems from its ability to contribute to the pathology of diseases including cancer [13, 14], atherosclerosis, rheumatoid arthritis, inflammatory bowel disease and osteoarthritis [15–21].

Vascular endothelial growth factor regulation is tightly controlled, involving regulation at the levels of transcription, translation and post-translational modification. Transcriptional regulation of the VEGF family members involves multiple transcription factors, including SP-1, AP-2, Egr-1, p53, TCF and HIF-1α [22], which directly interact at specific binding sites on the promoter sequence. VEGF is also known to induce STAT proteins such as STAT1 and STAT6 tyrosine phosphorylation and nuclear translocation [23]. Our current knowledge of VEGF regulation suggests that therapeutic modulation of VEGF transcription may represent a promising strategy in the battle against inflammation, cancer and possibly other immune disorders.

We have previously characterized a transcription factor named LPS-induced TNF-α factor (LITAF) that can form a complex with another transcription factor, STAT6B, to regulate inflammatory cytokines. Upon activation, this complex translocates from the cytosol to the nucleus, where it binds to promoters and up-regulates the transcription of multiple cytokines including TNF-α, IL-1α, MCP-2, RANTES and IL-10, in response to LPS stimulation [24]. We reported that LITAF phosphorylation and nuclear translocation was mediated by p38α [25]. LITAF has been demonstrated to play an important role in rheumatoid arthritis, Crohn's disease, innate immune dysregulation in the central nervous system and inflammatory changes in mesenteric fat linked to metabolic syndrome in obesity and insulin resistance [26]. Its role in mediating various inflammatory responses has led us to investigate whether LITAF/STAT6B is involved in the regulation of VEGF expression.

In this study, we report the identification of DNA binding domains important in LITAF and/or STAT6B-mediated transcriptional regulation of VEGF. In addition, VEGF in mouse macrophages or endothelial cells induces p38α phosphorylation which consequently activates both LITAF and STAT6B nuclear translocation. Finally, VEGF164-soaked matrigel implants placed subcutaneously in LITAF-deficient animals and in wild-type mice silenced for STAT6B exhibited significantly reduced VEFG-induced vessel formation in whole-body LITAF-deficient mice (TamLITAF^−/−^) as well as in STAT6B-silenced wild-type animals compared with respective control animals (wild-type animals or scramble siRNA-treated animals). The present data will be useful in the search for targets in the treatment of VEGF diseases.

## Materials and methods

### Cells and bacteria

U2OS cells (HTB-96, ATCC), RAW 264.7 cells (TIB 71, ATCC), endothelial cells (CRL-2280, ATCC), or mouse peritoneal macrophages from macrophage-specific LITAF-deficient mouse (macLITAF^−/−^, our laboratory) were cultured in DMEM media (Invitrogen, Grand Island, NY, USA) with 10% foetal bovine serum (FBS; Amresco, Inc., Solon, OH, USA) at 37°C in 5% CO_2_ atmosphere. Top 10 cells (Invitrogen) were used for the DNA construction according to the manufacturer's recommendations.

### Mice

Macrophage-specific LITAF-deficient mouse (macLITAF^−/−^) used for promoter assay, whole-body LITAF-deficient mouse (TamLITAF^−/−^) used for angiogenesis, and wild-type (WT) mice were generated by our laboratory as described previously [25, 27]. All mice protocols used in this study were approved by Boston University Animal Care and Use Committee. Mice used in experiments were 8–12 weeks of age, and were kept under strict specific pathogen-free (SPF) conditions. All procedures involving animals were approved by the Institutional Animal Care and Use Committee at Boston University Medical Center.

### Plasmid DNA constructs

The 630 bp mLITAF (GenBank Accession no. NM_019980) in-frame full-length cDNA fragment (mLITAFWT) was amplified by PCR using a primer pair (Table 1) [1]. The PCR product of mLITAF WT was electrophoresed and extracted from agarose, then subcloned into a vector of pcDNA3HA [24]. Four 3′-deletion sequences of mLITAF cDNA were generated by PCR with three specific 3′-reverse primers, and then subcloned into the vector pcDNA3HA. Products were named mLFR529 (1–529 bp), mLFR409 (1–409 bp) and mLFR289 (1–289 bp), as described in Table 1. The mouse STAT6B (GenBank Accession no. NM_009284) in-frame cDNA fragment (mSTAT6BWT) was amplified by PCR using a primer pair, and then subcloned into the pcDNA3HA after purification [2]. The eight diversified DNA deletions of mSTAT6B that were generated by PCR using designed primer pairs and subcloned into the pcDNA3HA vector were presented as m6BR547 (1–546 bp), m6BR397 (1–396 bp), m6BR247 (1–246 bp), m6BF246 (247–564 bp), m6BF396 (397–564 bp), m6BIa (163–396 bp), m6BIb (205–396 bp) and m6BIs (247–396 bp; Table 1). The mouse VEGF promoter DNA fragment (−394–54 bp) was produced by PCR from the mouse VEGF DNA clones (OPEN BIOSYSTEMS Clone ID 6816435) using a primer pair (Table 1) [3]. The amplified DNA fragment was purified and subcloned into a luciferase reporter vector pGL3-basic (mV-PWT). Six sequentially deleted mouse VEGF promoter DNAs were amplified by PCR with appropriate primer pairs and subcloned individually into the luciferase reporter vector pGL3-basic to generate the constructs as mV-P282 (−394 to −112 bp), mV-P189 (−394 to −205 bp), mV-P89 (−394 to −305 bp), mV-PI193 (−306 to −112 bp), mV-PI100 (−338 to −237 bp) and mV-PF/39 (−338–291 bp) (Table 1).

**Table 1:** Primer Pairs for DNA Construction

PCR product name	Primer pair	

	Sense	Antisense
mLITAF		
mLITAFWT	5′-gccaccatggtctctaacact-3′	5′-ctacaagcgcttgtaggt-3′
mLFR529	5′-gccaccatggtctctaacact-3′	5′-ctaaacgcatcccagcagaca-3′
mLFR409	5′-gccaccatggtctctaacact-3′	5′-ctaatagaaggagacaggctg-3′
mLF289	5′-gccaccatggtctctaacact-3′	5′-ctaaatgagccctgtggctgg-3′
mSTAT6B		
mSTAT6BWT	5′-gccaccatggcccgacggaacc-3′	5′-tcaaagcactaccagcccctg-3′
m6BR547	5′-gccaccatggcccgacggaacc-3′	5′-tcactgacctacccactgtcc-3′
m6BR397	5′-gccaccatggcccgacggaacc-3′	5′-tcagggaggtggaaaaggtg-3′
m6BR247	5′-gccaccatggcccgacggaacc-3′	5′-tcaagttccagcccacgcttg-3′
m6BF246	5′-gccatgggctgctctgattcc-3′	5′-tcaaagcactaccagcccctg-3′
m6BF396	5′-gccatgttcctccctaacccc-3′	5′-tcaaagcactaccagcccctg-3′
m6BIa	5′-gccatgggatcttgctcagct-3′	5′-tcagggaggtggaaaaggtg-3′
m6BIb	5′-gccatgggaggaggctttccg-3′	5′-tcagggaggtggaaaaggtg-3′
m6BIs	5′-gccatgggctgctctgattcc-3′	5′-tcagggaggtggaaaaggtg-3′
mVEGF promoter		
mV-PWT	5′-cgggattgcacggaaacttttcgt-3′	5′-atggtggaggtacagcagtaa-3′
mV-P282	5′-cgggattgcacggaaacttttcgt-3′	5′-gaggcccgggccggggcctgg-3′
mV-P189	5′-cgggattgcacggaaacttttcgt-3′	5′-ctcgagccgagcgcccactgcggc
mV-P89	5′-cgggattgcacggaaacttttcgt-3′	5′-ctcgagcgatcggtttgtctcctg-3′
mV-P193	5′-gagctgggagaagtgcta-3′	5′-gaggcccgggccggggcctgg-3′
mV-P100	5′-agaggggaggaagagaag-3′	5′-ctgtctgcgcacacggcc-3′
mV-PΔ39	5′-cgggattgcacggaaacttttcgt-3′	5′-ggcccgagctagcacttctccctactacggagcgag-3′
	5′-ggagaagtgctagctcgggcctggagaagccggggc-3′	5′-atggtggaggtacagcagtaa-3′

### Luciferase assay for promoter activities and gene silencing

With optimized reconstructed vector concentrations, the U2OS cells were transiently transfected with 50 ng/ml of pGL3-basic containing appropriate mVEGF promoter-reporter DNA alone or cotransfected with pcDNA3 constructed with individual 50 ng/ml samples of mLITAF WT cDNA or its deletions, including mLFR529, mLFR409, mLFR289 or mLFR169. The 100 ng/ml of reconstructed pcDNA3 vector containing inserts of mSTAT6B cDNA or its specific deletions including m6BR547, m6BR397, m6BR247, m6BF246, m6BF396, m6BFIa, m6BIb or m6BIs was cotransfected with 50 ng/ml of VEGF/pGL3-basic containing truncated mVEGF promoter-reporter DNAs as mV-PWT, mV-P282, mV-P189, mV-P89 or mV-P193 into the U2OS cells.

The U2OS cells were also transfected with mV-PWT/pGL-3 or any one of its truncated promoter DNAs, mSTAT6B/pcDNA3 and mLITAF/pcDNA3. As controls, U2OS cells were also cotransfected with unconstructed 100 ng/ml of pcDNA3, plus 100 ng/ml or 50 ng/ml of pGL3-basic with VEGF promoter insert. To silence mLITAF genes, U2OS cells were cotransfected with 100 ng/ml DNA of mSTAT6B (m6B) and 50 ng/ml of mLITAF, then treated with the siRNAs by HiPerFect (Qiagen, Valencia, CA, USA). After an overnight incubation at 37°C, the cells were washed in PBS and lysed in the luciferase cell lysis buffer. Luciferase activity was quantified as described above. Triplicate assays were performed and the data were analysed statistically.

### Chromatin immunoprecipitation (ChIP) assay

The pre-cultured 70–80% confluent RAW 264.7 cells were treated with 0.1 μg/ml *E. coli* LPS (Invitrogen) for 2 hrs, washed with PBS, and then cultured overnight. Cells were fixed in 0.5% formaldehyde/PBS. The chromatin was sheared using a ChIP-IT Express Enzymatic kit (Active Motif, Carlsbad, CA, USA). The 10 μg nuclear extracts (NE) from the cross-linked cells were immunoprecipitated with 1 μg antibody of anti-mLITAF, anti-mSTAT6B, or 1 μg normal IgG as control for 4 hrs at 4°C. DNA from each experimental group (IP) was isolated by elution, reverse cross-linking and Proteinase K treatment according to the manufacturer's instructions. The DNA was used as a template to perform PCR with primer pairs 5′-CGGGATTGCACGGAAACTTTTCGT-3′ and 5′-CCAGCTCCGATCGGTTTGTCT-3′ for −400/−300, 5′-CGGGATTGCACGGAAACTTTTCGT-3′ and 5′-CTGAGAGCCGAGCGCCCACTG-3′ for −400/−200, and 5′-CGGGATTGCACGGAAACTTTTCGT-3′ and 5′-CTCCCTTCTGGAACCGAGGCC-3′ for −400/−100. GAPDH primer pairs (Invitrogen) were used as a negative control. The ChIP assay was analysed using PCR and gel electrophoresis. A special ChIP was used with some modifications for Figure 3D. RAW 264.7 cells were transfected with DNAs overnight. Cells were fixed in 0.5% formaldehyde/PBS. The chromatin was sheared using a ChIP-IT Express Enzymatic kit (Active Motif). The 10 μg protein extracts from the cross-linked cells were immunoprecipitated with 1 μg antibody of anti-HA or 1 μg normal IgG as control for 4 hrs at 4°C. DNA from each experimental group (IP) was isolated and used to perform PCR according to the manufacturer's instructions.

### Western blot analysis

Mouse peritoneal macrophages from macrophage-specific LITAF-deficient mouse (macLITAF^−/−^), mouse endothelial cells, or U2OS cells were treated and cultured overnight. Whole-cell protein or nucleus protein from these pre-treated cells were purified with a commercial kit (Cat#78833; PIERCE Biotechnology, Rockford, IL, USA) following manufacturer's instructions. Proteins were equally loaded according to protein concentration and run in an SDS-PAGE gel. Protein bands were transferred to a membrane, then blotted with antibodies against mouse p-38α (sc-535; Santa Cruz, CA, USA), p-p-38α (sc-7973; Santa Cruz), LITAF (Cat# 611614; BD Biosciences, San Jose, CA, USA), STAT6B (5278; BioSynthesis, Inc., Lewisville, TX, USA), TBP (sc-34862; Santa Cruz), or actin (sc-1615; Santa Cruz) and tubulin (sc-58666; Santa Cruz) as control for Western blot analysis.

### siRNAs

The sequences of siRNA were designed by siRNA Wizard v3.1 (InvivoGen, San Diego, CA, USA) software based on mouse LITAF cDNA or mouse STAT6B cDNA. siRNA sense and their corresponding antisense strands were synthesized (Qiagen). The sequence of siRNA was labelled as follows (its function tested in this study attached): aaa[1] mLFsiRNA#1: 5′-GAATGAATCCACCTTCGTACT-3′ (significant knockdown of mouse LITAF expression) [2] mLFsiRNA#2: 5′-AATGAATCCACCTTCGTACTA-3′ (significant knockdown of mouse LITAF expression); [3] m6BsiRNA#1: 5′-GATGTCACTCCCTATTTCATA-3′ (significant knockdown of mouse STAT6B expression), [2] m6BsiRNA#2: 5′-GAGCACTCCATGGCTGTCTTT-3′ (no effect on mouse STAT6B and other related genes); [4] siRNAControl (Cat#1027280; Qiagen).

### ELISA

Mouse endothelial cells were treated with the siRNAs (40 nM mLFsiRNA#1, 40 nM m6BsiRNA#1 or 40 nM siRNAControl as control) for overnight. Cells were treated with 0.2 μg/ml LPS for 3 hrs. The conditioned media from cells were collected and subjected to ELISA for detection of endogenous expression of VEGF (Invitrogen). ELISA immunoreactivity was quantified using a macroplate reader (Model 680; Bio-Rad, Hercules, CA, USA). Total protein concentration of corresponding cell lysate was measured and used for normalization.

### Real-time PCR

To *in vitro* determine the effect of VEGF164 treatment on cells, mouse peritoneal macrophages from LITAF−/− or WT mice were cultured for 3 days. Cells were treated with 50 ng/ml VEGF164 for 2, 8 hrs or untreated as control. Total RNA from cells was isolated using RNeasy Mini Kit (Cat#74104). To determine the effect of VEGF164 treatment *in vivo*, four groups of mice were used: WT, LITAF-deficient, WT injected subcutaneously with either 6BsiRNA#1 or with 6BsIRNA#2 as control. All mice were implanted with VEGF164-infused Matrigel for 10 days. Matrigel implantation from each test was dissected and their total RNAs were purified by RNeasy Midi Kit (Cat#: 75142; Qiagen). Total RNAs purified above were subjected to RT-PCR using RT-PCR kits (iScript & iQ SYBR Green, Bio-Rad) with VEGF primer pairs 5′-atgaactttctgctctcttggg-3′ and 5′-tcaccgccttggcttgtcaca-3′ or β-actin (housekeeping gene) primer pairs 5′-gctccggcatgtgcaa-3′ and 5′-aggatcttcatgaggtagt-3′, following the manufacturer's instructions.

### *In vitro* chemotaxis assay

The pre-cultured endothelial cells (1 × 10^6^ cells) were untreated as control or treated with the siRNAs (40 nM mLFsiRNA#1 and/or 40 nM m6BsiRNA#1) overnight. Cells were treated with 50 ng/ml VEGF164 for 24 hrs. Cells were collected, stained with WST-1 kit (Cat#: k304-2500; BioVision Inc., Milpitas, CA, USA), and used for cell migration by chemoTx Disposable Chemotaxis System kit (Cat# 116-8; Neuro Probe, Inc, Gaithersburg, MD, USA) following the manufacturers' instructions.

### VEGF-infused Matrigel angiogenesis assay

For angiogenesis assay, four groups of mice were used: WT mice (control group); TamLITAF^−/−^ (test group), m6BsiRNA#1 knock-down WT mice (test group) and m6BsiRNA#2-treated WT mice (scramble siRNA control group). For siRNA-treated mice, WT mice were injected subcutaneously with 40 μM siRNA every other day for the first 8 experimental days. All mice were age- and weight matched. In this assay, VEGF164 (recombinant mouse VEGF Cat #493-MV-005/CF; R&D Systems, Inc., Minneapolis, MN, USA) was incorporated into cold liquid Matrigel (BD Bioscience) at a concentration of 50 μg/ml, and 0.5 ml VEGF-infused Matrigel were implanted subcutaneously in the rectus abdominus of all group mice. Under physiological conditions, after subcutaneous injection, the Matrigel solidifies and permits penetration by host cells and the formation of new blood vessels. Mice were killed 10 days after the Matrigel implantation and the Matrigel plugs were dissected and examined for vascular density. Dissected specimens were fixed overnight in 4% paraformaldehyde (PFA, USB) in PBS, frozen in OCT/sucrose (1:1 vol/vol), and sectioned using a cryostat. Sections were stained with anti-CD31 (BD Phamagen, Franklin Lakes, NJ, USA), a well-established marker for angiogenic activity, and counterstained. Vascularization was then quantified on stained sections by histomorphometric analysis.

### Statistical analysis

Continuous data are conveyed as mean ± SEM. Luciferase activities and ELISA were assayed in triplicates. Measurements in Matrigel angiogenesis assays were made by two independent evaluators. Multiplicity of comparisons to the control group was performed by anova. A *P* < 0.05 was considered statistically significant.

## Results

### Effects of mLITAF/mSTAT6B on VEGF promoter activity

**Fig. 1:**
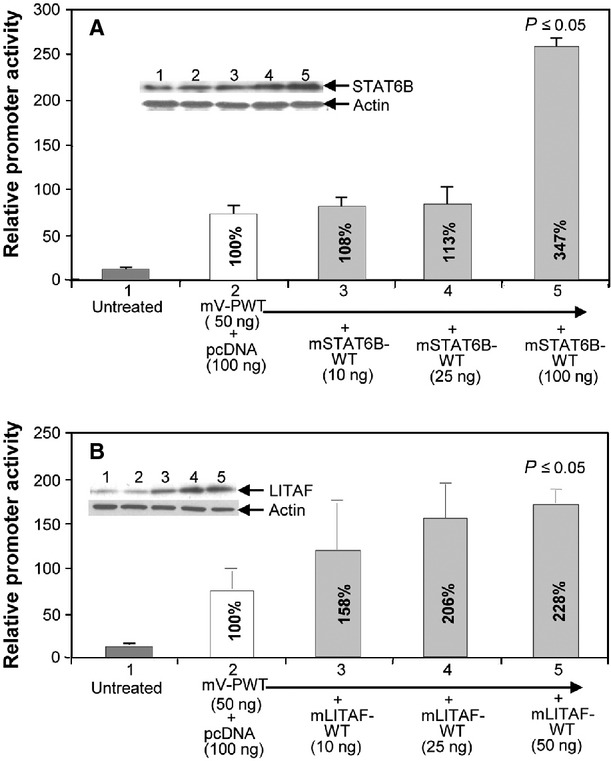
Effect of mSTAT6B and mLITAF on regulation of VEGF promoter activity. 1 x 10^5^ pre-cultured U2OS cells were cotransfected with DNA of 50 ng/ml mV-PWT (#s 2-5) plus 100 ng of pcDNA as control or plus different concentrations (10 ng, 25 ng or 100 ng) of mSTAT6BWT (**A**) or mLITAFWT (**B**) for 8 hrs. Lysate from each experimental cell group was analysed. Top Panel: Western blot assay (#s 1–5) using antibodies against actin as control or mSTAT6B (**A**) or mLITAF (**B**). Lower panel: luciferase assay of VEGF promoter activity. Triplicate assays were conducted. The relative promoter activities were analysed and graphed. Mean SEM.

Overexpression assays using a dose-course study were performed to assess the effects of mLITAF or mSTAT6B on the VEGF promoter activity. A quantity of 50 ng mV-PWT DNA was cotransfected into U2OS cells at different concentrations of mSTAT6B (Fig. 1A) or mLITAF (B). The lysate from each experimental group was used for the luciferase assay. As shown in Figure 1A, promoter activity by cotransfection of mV-PWT DNA with pcDNA was used as a 100% baseline for comparison of the effect of subsequent transfections. At concentrations of 10 and 25 ng of mSTAT6B, there were no significant changes in VEGF promoter activity. However, at concentrations of 100 ng of STAT6B, the relative VEGF promoter activity was 3.4-fold greater relative to the baseline. VEGF promoter activity after 50 ng mLITAF cotransfection was almost 2.2 times greater than the activity of the baseline (Fig. 1B).

### Analysis of promoter and protein regions involved in VEGF promoter activity

**Fig. 2:**
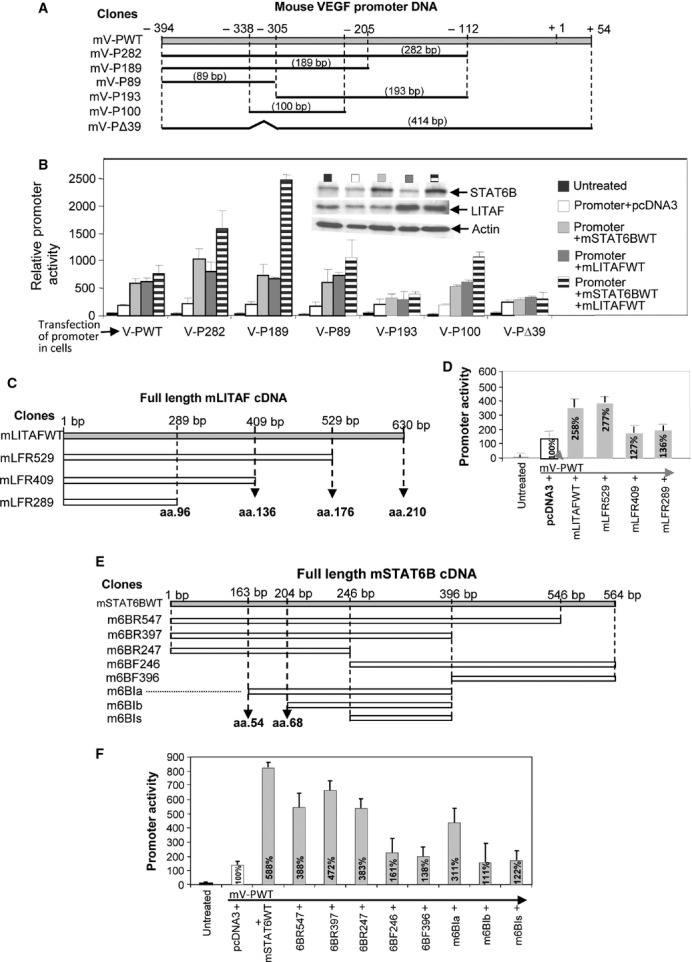
Detection of mLITAF- or mSTAT6B-mediated VEGF promoter activity by mutagenesis. Diagram of mouse WT VEGF promoter DNA and its deletions (**A**), of mouse WT LITAF cDNA and its deletions (**C**) and of mouse WT STAT6B cDNA and its deletions (**E**). (**B**) 1x10^5^ pre-cultured U2OS cells were cotransfected with 50 ng/ml mV-PWT DNA or its deletions plus pcDNA as control, mLITAF or mSTAT6B overnight. Lysate from each experimental cell group was analysed. Top Panel: Western blot assay using antibodies against actin as control, mSTAT6B or mLITAF. Lower panel: luciferase assay of VEGF promoter activity. (**D**) Cells were cotransfected with mV-PWT DNA plus pcDNA as control, mLITAFWT or its varied deletions. (**F**) Cells were cotransfected with mV-PWT DNA plus pcDNA as control, mSTAT6BWT or its varied deletions overnight. The lysate from each experimental cell group was analysed by luciferase assay. Triplicate assays were conducted. Mean SEM.

To determine the region of the VEGF promoter important for mLITAF or mSTAT6B binding activity, the VEGF promoter was truncated and promoter assays were performed. The VEGF promoter region was divided into six different sized DNA fragments (Fig. 2A), and its promoter activities were further analysed. As shown in Figure 2B, the white bar in each group was used as baseline for comparison. In every group, VEGF promoter activity was up-regulated by the overexpression of mLITAF (light grey bar), mSTAT6B (dark grey bar), or mLITAF/mSTAT6B together (dark horizontal bar), except in groups V-P193 and VPΔ39, compared with the baseline group. V-PI93 and V-PΔ39, two fragments that did not contain the −338 to −305, were not affected by the overexpression of mLITAF and mSTAT6B, suggesting that this region is important to the binding activity of mLITAF and mSTAT6B. To evaluate the mLITAF protein domain important to VEGF promoter activity, mutagenesis experiments were performed, with the full-length mLITAF cDNA mutated into four deletions (Fig. 2C), and its biological functions examined. As shown in Figure 2D, the second bar labelled mV-PWT was used as baseline for comparison. VEGF promoter activity was strongly up-regulated by the overexpression of mLFWT and mLFR529 except mLFR409/mLFR289 which did not contain the amino acid (aa) residues downstream of 136, suggesting that in this domain aa 136–176 may be important to the regulation of VEGF promoter activity.

To determine the mSTAT6B protein domain that is important to VEGF promoter activity, the protein was truncated. The mSTAT6B cDNA was divided into eight different sized DNA fragments, as demonstrated in Figure 2E, and its biological functions were further analysed. As shown in Figure 2F, the second bar labelled mV-PWT was used as baseline for comparison. VEGF promoter activity was up-regulated by the transfection of m6BR547, m6BR397, m6BR247 or m6BIa, but transfection of m6BF246, m6BF396, m6BIb or m6BIs did not significantly regulate VEGF promoter activity seemingly due to the lack the aa residue from 54 to 68, that may be an important domain to the regulation of VEGF promoter activity.

### Interaction between mLITAF binding domain and VEGF promoter

**Fig. 3:**
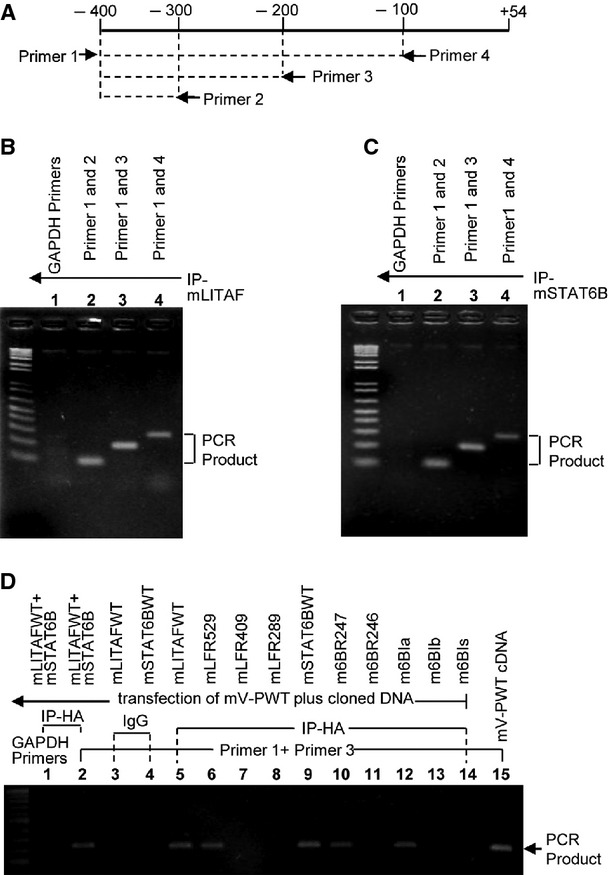
Chromatin immunoprecipitation (ChIP) Assay. RAW 264.7 cells were treated with 0.1 μg/ml *E. coli* LPS for 2 hrs, washed with PBS, then cultured overnight. ChIP was carried out using a ChIP-IT Express Enzymatic Kit. Diagram arrows indicate the location of primer pairs in mV-PWT promoter DNA (**A**). The genomic template DNA from anti-mLITAF precipitated (**B**) or anti-mSTAT6B precipitated (**C**) nuclear extracts (NE) of cells was used to amplify VEGF promoter DNA with 3 VEGF primer pairs or GAPDH primer pairs as control by PCR. The PCR products are indicated with arrows. (**D**) Determination of DNA-protein binding site. RAW 264.7 cells were cotransfected with mV-PWT plus mLITAF (#s 1-3,5), mSTAT6B (#s 1,2,4, & 9), mLITAF deletions (#s 6-8), or mSTAT6B deletions (#s 10-14). The DNA from anti-HA- (#s 1 & 2, 5-14), or from anti-IgG- (as control, #s 3 & 4) precipitated protein extracts of cells or mV-PWT cDNA as the positive control (#15) was used to amplify VEGF promoter DNA with VEGF primer pairs (primer1+primer3, #s 2-15) or with GAPDH primer pairs as control (#1) by PCR. The PCR products (200 bp) are indicated with arrows.

Once the DNA binding sequence on the VEGF promoter region was identified, we were interested in assessing protein–DNA interactions between VEGF promoter and mLITAF protein or mSTAT6B protein. ChIP analysis assays using LPS-treated RAW 264.7 cells were performed. Three primer pairs were designed (Fig. 3A) almost covering the VEGF promoter region from −338 to −305 (Fig. 2A) and a ChIP assay was performed (Fig. 3B and C). The DNA fragment of VEGF promoter was amplified by PCR with both IP-mLITAF (Fig. 3B) and IP-mSTAT6B (Fig. 3C), compared with the negative control (GAPDH). The region from −338 to −305 of the VEGF promoter appeared to contain a binding site for mLITAF and mSTAT6B. To examine whether the region of mLITAF aa 136–176 or mSTAT6B aa 54–68 is the important domain for interaction with VEGF promoter DNA, a modified ChIP assay was further performed. As shown in Figure 3D, no PCR amplification of the VEGF promoter DNA was observed when cells were transfected with DNA lacking mLITAF (mLFR289) aa 136–176 or mSTAT6B (m6BR246, m6Bib, or m6BIs) aa 54–68, confirming the results above.

### Down-regulation of VEGF promoter activity by silencing mLITAF and mSTAT6B activity

**Fig. 4:**
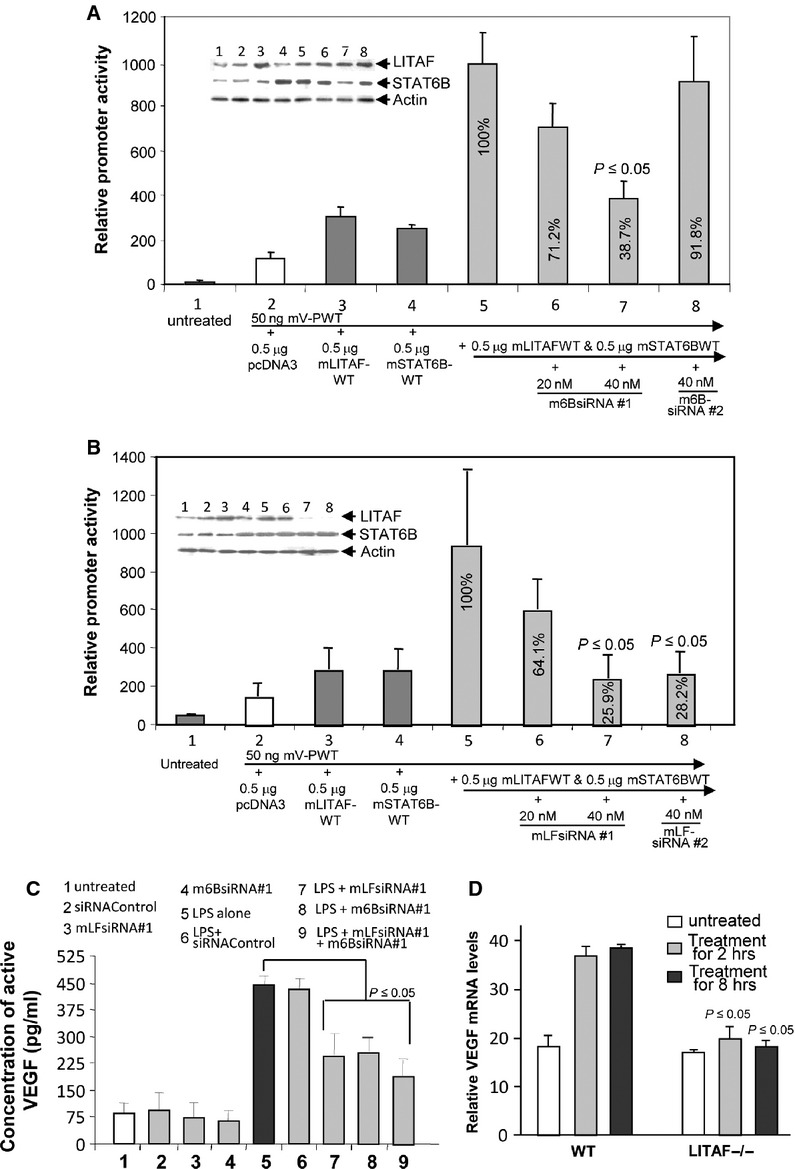
Analysis of VEGF promoter activity and gene expression after knockdown of mLITAF and/or mSTAT6B in cells. (**A**) U2OS cells were untreated as control (group 1), or cotransfected with 50 ng mV-PWT DNA (group 2–8) plus 0.5 μg pcDNA as control (group 2), 0.5 μg mLITAFWT (group 3), 0.5 μg mSTAT6BWT (group 4), or both 0.5 μg mLITAFWT and 0.5 μg mSTAT6BWT (groups 5–8), then combined with 20 nM m6BSsiRNA#1 (group 6), 40 nM m6BsiRNA#1 (group 7), or 40 nM m6BsiRNA#2 (group 8) overnight to test the efficacy of the siRNAs. Lysate from each experimental cell group was analysed. Top panel: Western blot assay using antibodies against actin as control, mSTAT6B or mLITAF. Lower panel: luciferase assay of VEGF promoter activity. (**B**) U2OS cells were untreated as control (group 1), or cotransfected with mV-PWT DNA (group 2–8), plus either 0.5 μg pcDNA as control (group 2), 0.5 μg mLITAFWT (group 3), mSTAT6BWT (group 4), or both mLITAFWT and mSTAT6BWT (groups 5–8), then combined with 20 nM mLFsiRNA#1 (group 6), 40 nM mLFsiRNA#1 (group 7), or 40 nM mLFsiRNA#2 (group 8) overnight. Lysate from each experimental cell group was analysed. Top Panel: Western blot assay using antibodies against actin as control, mSTAT6B or mLITAF. Lower panel: luciferase assay of VEGF promoter activity. (**C**) Mouse endothelial cells were untreated as control (group 1), or treated with siRNA (groups 2–4, 6–9) overnight and further treated with LPS (groups 5–9) for 3 hrs. ELISA immunoreactivity was quantified and graphed. Intensity in ELISA from LPS alone-treated cells as positive control was assigned to a base value (100%). Intensity of other treatments (LPS+siRNA) was calculated relative to this base value. (**D**) Mouse peritoneal macrophages (wild-type as control or macrophage-specific LITAF-deficient mouse, macLITAF^−/−^) were untreated as control (white bars) or treated with 50 ng/ml VEGF164 for 2 (grey bars) or 8 hrs (black bars). Total mRNA from treated cells was assessed by RT-PCR and normalized with β-actin. Intensity of VEGF mRNA from VEGF164-treated cells for 8 hrs was assigned to a base value (100%). Intensity of VEGF mRNA from other treatments was calculated relative to this base value. Triplicate assays above were conducted. Mean SEM.

To determine if either or both mLITAF or mSTAT6B participate in regulating the VEGF promoter, gene knockdown by siRNAs was performed. As shown in Figure 4A, VEGF promoter activity was significantly inhibited (60%) when mSTAT6B was knocked down with m6BsiRNA#1 at a concentration of 40 nM (group 7), compared with the positive control (group 5). Treatment with m6BsiRNA#2 (negative control) did not knock down mLITAFWT-mSTAT6B-mediated VEGF promoter activity (group 8). VEGF promoter activity was also strongly reduced by 25–28% at a concentration of 40 nM in both mLFsiRNA#1 (Fig. 4B, group 7) and mLFsiRNA#2 (Fig. 4B, group 8) compared with the baseline (Fig. 4B, group 5), suggesting that silencing mLITAF and mSTAT6B gene expression by siRNAs down-regulates VEGF promoter activity. To identify whether mLITAF/mSTAT6B are involved in VEGF gene expression, mLITAF and/or mSTAT6B were knocked down by siRNA in mouse endothelial cells and LPS-induced VEGF endogenous protein was assessed by ELISA. As shown in Figure 4C, knockdown of mLITAF and/or mSTAT6B (group 7–9) in wild-type mouse endothelium cells significantly reduced LPS-induced VEGF production from 57% to 43% compared with the positive control (group 5).

To further determine the effect of LITAF deficiency on VEGF transcription, RT-PCR was performed. As shown in Figure 4D, VEGF164-induced VEGF mRNA levels for 8 hrs in LITAF- knockout cells was decreased to 47% compared with the positive control (VEGF164-treated wild-type cells as 100% baseline). Overall, mLITAF- and/or mSTAT6B-deficiency were found to be involved in down-regulating VEGF gene expression.

### Effects of VEGF on LITAF/STAT6B translocation

**Fig. 5:**
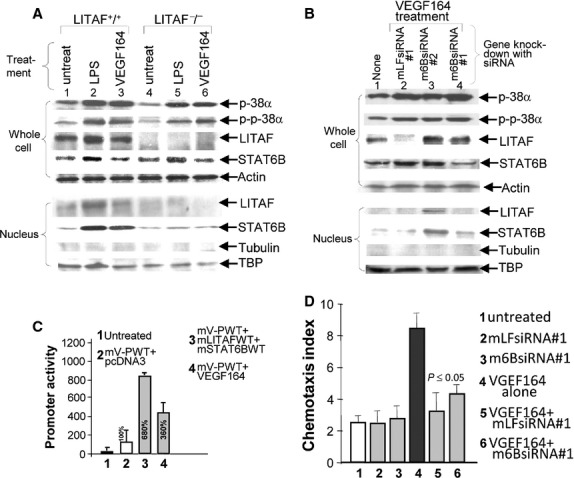
Analysis of VEGF164-induced protein nuclear translocation and cell migration in the presence/absence of mLITAF/mSTAT6B. (**A** & **B**) Protein separated and purified from LPS- or VEGF164-treated mouse peritoneal macrophages (macrophage-specific LITAF-deficient mouse, macLITAF^−/−^ or wild-type cells as control, (**A**), or proteins from siRNA & VEGF164 cotreated wild-type mouse endothelial cells (5B) were detected by Western blotting with antibodies against mouse p38α, p-p-38α, LITAF, STAT6B, or actin, tubulin and TBP as control. (**C**) Mouse peritoneal macrophages were untreated as control (group 1), or cotransfected with 50 ng mV-PWT DNA (groups 2–4) plus either 0.5 μg pcDNA as control (group 2), or plus both 0.5 μg mLITAFWT and 0.5 μg mSTAT6BWT (group 3), or plus 50 ng/ml VEGF164 (group 4) overnight. Cells were analysed by luciferase assay. (**D**) Measurements of cell migration by *in vitro* chemotaxis assay. 1x10^6^ pre-cultured mouse endothelial cells were untreated as control (group 1), or treated with siRNA (groups 2, 3, 5 & 6) overnight and then treated with VEGF164 (groups 4–6) for 24 hrs. The treated cells were used for cell migration assay. Triplicate assays were done. The measurement was graphed.

To determine whether VEGF secretion can affect p38α-mediated LITAF and/or STAT6B expression, Western blot analysis was performed. Treatment of both LPS and VEGF164 in mouse peritoneal macrophages from WT mouse induced p38α production and its phosphorylation which in turn activated LITAF or STAT6B nuclear translocation (Fig. 5A, lanes 2 and 3). However, phosphorylated p38α could not induce STAT6B translocation in the absence of LITAF (Fig. 5A, lanes 5 and 6). The same mechanism was observed in endothelial cells (Fig. 5B) namely siRNA-knockdown of LITAF (Fig. 5B, lane 2) or STAT6B (Fig. 5B, lane 4) with mLFsiRNA#1 or m6BsiRNA#1 significantly reduced LITAF and STAT6B expression leading to a lack of LITAF or STAT6B translocation into the nucleus of cells. To examine whether treatment of macrophages with VEGF164 can trigger VEGF promoter activity (autocrine function), a luciferase assay was performed. As shown in Figure 5C, VEGF164-treated cells (group 4) increased 3.6 fold in promoter activity compared with the control (group 2), demonstrating that VEGF secretion can in turn induce VEGF promoter activity. To test whether knock-down mLITAF/mSTAT6B is involved in VEGF164-induced cell migration, a chemotaxis assay was performed. As shown in Figure 5D, VEGF164-induced migration was decreased to 38% (group 5) in mLITAF knock-down cells and to 50% in mSTAT6B knock-down cells (group 6) compared with the positive control (treated wild-type cells as 100% baseline, group 4).

### *In vivo* angiogenesis assay

**Fig. 6:**
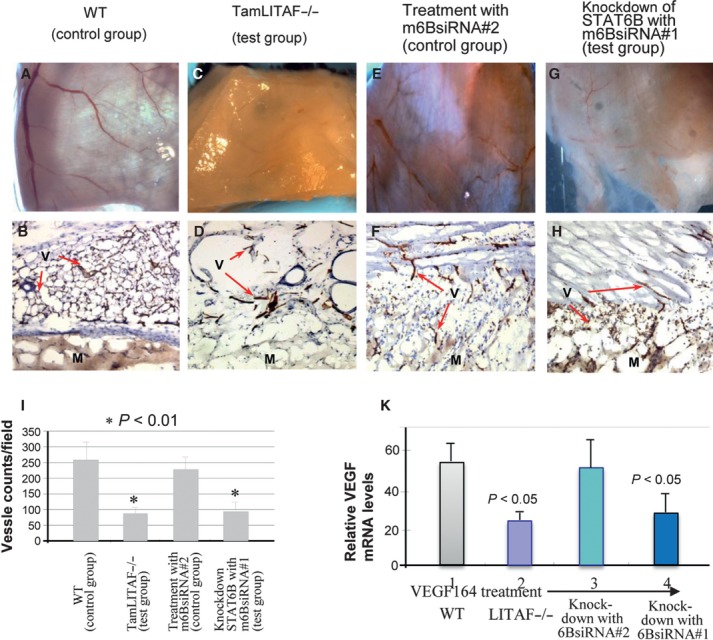
LITAF or STAT6 deficiency affects VEGF-induced angiogenesis *in vivo*. Photographs of VEGF-soaked Matrigel by surgical microscopy excised from wild-type (**A**), TamLITAF KO (**C**), mSTAT6B siRNA#2 (**E**) and mSTAT6B siRNA #1 (**G**) mice after 10 day implantation (x10). Corresponding plugs were prepared and stained with anti-CD31 for vessel staining: wild-type (**B**), mLITAF KO (**D**), mSTAT6B siRNA#2 (**F**) and mSTAT6B siRNA#1 (**H**). Arrows point at blood vessels stained on the Matrigel. This experiment is representative of two individual studies (3 mice per group). (**I**): Vessel density: 10 random high-power fields per 5 Matrigel sections were evaluated and stained capillary blood vessels were quantified by histomorphometric analysis (*, p < 0.01): M, matrigel; V, vessels. (**K**) Total mRNA from treated tissue was assessed by RT-PCR and normalized with β-actin. Intensity of VEGF mRNA from VEGF164-treated WT tissue was assigned to a base value (100%). Intensity of VEGF mRNA from other treatments was calculated relative to this base value. Triplicate assays were conducted. Mean SEM.

To confirm these observations *in vivo,* a Matrigel angiogenesis assay was performed. VEGF164-soaked matrigels retrieved 10 days after implantation from experimental mice (TamLITAF^−/−^ or m6BsiRNA#1 knockdown) showed a significant reduction in vessel density with evidence of abundant blood vessels in the Matrigel plug (Fig. 6C, D, G and H) compared with the control mice (WT animals or m6BsiRNA#2-treated animals, Fig. 6A, B, E and F). Histomorphometric analysis of sections stained with an antibody against CD31, a biomarker for angiogenesis, confirmed that blood vessel density in control animals (WT or m6BsiRNA#2-treated mice) was three times higher than in TamLITAF^−/−^ or m6BsiRNA#1 knock-down mice (Fig. 6I, *P* < 0.05).

To further determine whether VEGF transcription is mediated in the absence of LITAF or in the silence of STAT6B, RT-PCR was performed. VEGF164 treatment led to a reduction in VEGF mRNA level by 43.6% in the LITAF-deficient animals (Fig. 6K, group 2), and by 50.5% in the STAT6B knock-down animals (Fig. 6K, group 4) compared with the positive control VEGF164-treated WT animals (100% baseline, group 1). Overall, mLITAF- and/or mSTAT6B deficiency were found to affect negatively VEGF gene expression.

## Discussion

In this study, we report the identification of DNA binding domains important in LITAF and/or STAT6B-mediated transcriptional regulation of VEGF. In addition, VEGF in WT mouse peritoneal macrophages or endothelial cells induces p38α phosphorylation, which then activates LITAF or STAT6B nuclear translocation. Finally, VEGF164-soaked Matrigel implants placed subcutaneously in LITAF-deficient animals or in wild-type mice silenced for STAT6B exhibited significantly reduced VEGF-induced vessel formation in TamLITAF^−/−^, as well as in STAT6B-silenced WT animals (m6BsiRNA#1 knock-down mice), compared with control animals (WT animals or m6BsiRNA#2-treated animals). Earlier we reported that LITAF and STAT6B, either alone or in a complex, mediate transcription of TNF-α in response to LPS induction [24]. Specifically, LITAF was identified as a regulator of transcription of inflammatory cytokines (TNF, MCP-1, IL-10), and STAT6B was recognized as a potential cofactor in LPS-stimulated macrophages highly homologous to STAT6 in the region from amino acids 151–404 but completely different in the region from amino acids 1–150 [25]. Locally elevated LITAF protein was reported in Crohn's disease (CD) and ulcerative colitis (UC), two major TNF-mediated inflammatory bowel diseases (IBD) [28]. CD and UC share some common pathological characteristics such as immune activation, leucocyte infiltration into tissues and increased vascular density possibly mediated by VEGF [29]. Indeed, Chidlow *et al*. found that when VEGF expression was inhibited using an siRNA, the pathological angiogenesis and inflammatory response was attenuated in CD4(+) CD45RB(high) T cell-dependent experimental colitis [30]. As a result, angiogenesis is thought to play a key role in the development of IBD where both an increase in the area of endothelium available for exchange and the blood constituents extravasated into surrounding tissue heighten the severity of the disease [31]. Targeting the mediators involved in VEGF gene regulation might be a novel way to inhibit angiogenesis in pathological conditions.

Our previous data indicate that LITAF and STAT6B are induced by *Porphyromonas gingivalis* LPS *via* TLR2 or by *E. coli* LPS *via* TLR4. Their production is MyD88 dependent. Subsequently, they are phosphorylated by p38α before protein–protein interactions form a complex. This complex in the cytoplasm translocates into the nucleus [25]. Our new findings here support this signalling pathway for angiogenesis: VEGF binds to a receptor (VEGFR1 or VEGFR2) in MyD88+/+ cells [32–34] and induces p38α phosphorylation [35]. Although p38α was reported to be involved in increasing VEGF-induced vascular permeability [36, 37], the role of p38α in the regulation of VEGF gene expression remains debatable. Our previous data [25] along with the data presented in this study show that treatment of mouse peritoneal macrophages with either LPS or VEGF164 induces p38α production and phosphorylation, which in turn activates LITAF and/or STAT6B nuclear translocation leading to an up-regulation of VEGF gene expression. This evidence linking p38/LITAF *in vitro* prompted us to test this hypothesis *in vivo* confirming the importance of LITAF and STAT6B in VEGF-induced angiogenesis.

To further understand protein–DNA interaction(s) between LITAF or STAT6B and VEGF, we performed sequential deletions of the VEGF promoter and truncated mLITAF and mSTAT6B sequences, which were then transiently multitransfected into U2OS cells. An increase in VEGF promoter activity, assessed by luciferase activity, was detected in the presence of overexpressed mLITAF and/or mSTAT6B. A 34 bp DNA region located between −338 and −305 within the VEGF promoter sequence was identified as a protein binding site for LITAF and STAT6B (Figs 2 and 3). On the basis of our chromatin immunoprecipitation (ChIP) assay data, we report that LITAF and STAT6B may work synergistically to up-regulate VEGF gene expression by binding to the same region of the VEGF promoter (−338 to −305), indicating that the DNA binding site in the VEGF sequence may be specific for both LITAF and STAT6B proteins in initiating transcription.

The early VEGF-blocking therapies in cancer clinical trials have been rather disappointing. The possible reason might be that when VEGF is blocked, the angiogenic process is maintained by the up-regulation of other growth modulators [38]. More interestingly, LITAF and TNF superfamily member 15 (TNFSF15) is up-regulated by 5′ adenosine monophosphate (AMP)-activated protein kinase (AMPK) in LNCaP and C4-2 prostate cancer cells. In conjunction, intratumoural injection of TNFSF15 significantly reduces the size of tumours and number of blood vessels. The regulatory axis of AMPK–LITAF–TNFSF15 even suggests that LITAF may function as a tumour suppressor [39]. Nevertheless, the angiogenesis assay in LITAF knockout mice exhibited a significant decrease in vessel density compared with the wild-type (Fig. 6), which suggests that angiogenic function of VEGF was thwarted in LITAF knockout mouse. Collectively, our recent investigation showed that the up-regulation of VEGF expression by the association of LITAF and STAT6B may play an important role in the inflammatory signalling pathway and benefit tumour development. While LITAF and STAT6B seem to work synergistically when tested by promoter assays (Fig. 4A and B) by ELISA, this synergism seems to be missing. This may be due to the fact that LPS treatment of cells induces VEGF expression not resulting only from LITAF/STAT6B binding activity but also from other factors. Indeed, LPS-induced VEGF production is decreased due to the knock-down LITAF or STAT6B, but this VEGF expression level remains 2–4-fold (Fig. 4C, group 7–9) higher than negative controls (Fig. 4C, group 1–4) possibly because of other factors.

In this study, we found that overexpression of mouse LITAF and/or STAT6B significantly up-regulated the gene expression of mouse VEGF *via* its binding activity. mLITAF and/or mSTAT6B deficiency showed a significant reduction in VEGF protein and its mRNA levels. We also found that siRNA-mediated knockdown of mLITAF and mSTAT6B inhibited VEGF expression and endothelial cell migration. A further *in vivo* assay indicated that the angiogenic function of VEGF is thwarted in LITAF knockout mice. Taken together, we conclude that LITAF and STAT6B play an important role in VEGF regulation and emphasize its potential as a therapeutic target in treating various VEGF-related diseases and inflammatory processes.
